# The impact of digital imaging in the field of cytopathology

**DOI:** 10.4103/1742-6413.48606

**Published:** 2009-03-06

**Authors:** Liron Pantanowitz, Maryanne Hornish, Robert A. Goulart

**Affiliations:** Department of Pathology, Baystate Medical Center, Tufts University School of Medicine, Springfield, MA, USA

**Keywords:** Cytology, cytometry, digital, image, informatics, Pap test, proficiency testing, screening, telecytology, virtual image

## Abstract

With the introduction of digital imaging, pathology is undergoing a digital transformation. In the field of cytology, digital images are being used for telecytology, automated screening of Pap test slides, training and education (e.g. online digital atlases), and proficiency testing. To date, there has been no systematic review on the impact of digital imaging on the practice of cytopathology. This article critically addresses the emerging role of computer-assisted screening and the application of digital imaging to the field of cytology, including telecytology, virtual microscopy, and the impact of online cytology resources. The role of novel diagnostic techniques like image cytometry is also reviewed.

## INTRODUCTION

A digital image is represented in a computer by a two-dimensional array of numbers (bitmap or raster image), each element of which represents a small square area of the picture, called a picture element (pixel). Such images can be transmitted or stored in a compressed form (reduced image size). Compression algorithms may be “lossless” (no loss of data) or “lossy” (some detail is lost).

Digital images can be created by a variety of input devices, such as a digital camera. The imaging process involves capture, saving (storage), editing (if necessary), and sharing (viewing, displaying, printing) digital images.[1] This process, as it relates to applications in pathology, has yet to be standardized.[2] In the field of cytology, digital images are used for telecytology, training, education (e.g. publications, conferences, and web pages), proficiency testing, and automated screening of Pap test slides.[3] In many ‘developed’ countries, pathologists are increasingly integrating digital images into their practice of medicine.[4–5] Image-enhanced reports are also a growing trend among pathology practices.[6] Therefore, several laboratory information system vendors are currently integrating digital image acquisition and storage modules into their products. Multiple digital images are usually needed to document cytologic features in each cytopathology case.[7] To date, there has been no systematic review on the impact of digital imaging to the practice of cytopathology. This review will discuss several digital imaging technologies currently available and analyze the literature specific to the field of cytopathology. The benefits and limitations of digital cytopathology are highlighted.

## TELECYTOLOGY

Telecytology, a component of the broader field of telepathology, is the practice of cytology at a distance, by using telecommunication to transmit digital images, often when the cytologist and slide (containing the patient's cytologic material) are separated by a distance. The emergence of technology that supports digital imaging, along with greater image quality and higher processing capacity of computers, has promoted the use of telecytology and telepathology.[8] Telepathology can be used for diagnosis, consultation, or education.[9–10] There have been several studies on telecytology [Table 1].[9,11–18] Nine studies from 1996 to 2007, in which sufficient data was recorded to report on concordance and interobserver variability, have been included in Table 1. Both gynecological specimens (i.e. Pap tests) and nongynecological cases have been shown to be amenable to telecytology.

**Table 1:** Publications reporting experience with telecytology (GYN = gynecological specimens [i.e. Pap tests]; Non-GYN = nongynecological specimens)

*Reference*	*Date*	*Country*	*Specimen*	*System*	*Concordance %*	*Interobserver variability*	*Limitations*
[11]	1996	USA	GYN	Static	60	Kappa = 0.20	Frequent undercalling of dysplasia
[12]	1998	Germany	GYN	Dynamic*	65	Kappa not recorded	Monolayers more problematic than conventional smears
[13]	1998	USA	Non-GYN	Static	67-91	Kappa not recorded	Insufficient images and poor image quality
[14]	2000	USA	Non-GYN	Static	80-96	Kappa > 0.6	Inexperience with the system
[15]	2001	USA	GYN	Static	Good (no % recorded)	Kappa 0.32 - 0.58	Poor reproducibility
[9]	2001	USA	Non-GYN	Static	69	Kappa not recorded	Poor quality images (out of focus)
[16]	2003	USA	Non-GYN	Static	Good to excellent	Kappa 0.22 - 0.556	Poor quality images with inability to focus on thick cellular groups
[17]	2004	Japan	GYN and non-GYN	Static	89	Kappa not recorded	Lengthy time (up to 20 minutes) to capture images
[18]	2007	Iran	Non-GYN	Static	89	Kappa = 0.71	Lengthy time (up to 30 minutes) to capture images and poor image quality

* Although a remotely controlled (dynamic) telecytology system was employed in this study, only preselected (static) areas were used.

Concordance is a measure of agreement between a telecytology diagnosis and that rendered after a review of the glass slide. Based upon these tabulated studies, it is apparent that while concordance improved over the years, it still remains far from perfect. Improvements were likely linked to advancing technology, user training, and/or familiarity with such systems. The diagnostic reproducibility of telecytology users is judged by kappa values (> 0.75 indicates excellent agreement, 0.58 – 0.74 good, 0.40 – 0.57 fair, and 0.20 – 0.39 poor reproducibility). Table 1 shows only modest agreement between observers, supporting the need for further training and perhaps proficiency testing in the use of telecytology.

Diagnostic accuracy (i.e. correct telecytology diagnosis correlating with the final pathology diagnosis) has only been recorded in a limited number of studies. Earlier studies found the accuracy of telecytology to be less than that of light microscopy.[11] In conjunction with technological advances, more recent studies have demonstrated improved accuracy. Therefore, it is not surprising that telecytology has been shown by some authors to be adequate for on-site rapid cytology diagnoses,[19] and may, therefore, effectively guide specimen triage. Accuracy in later studies has ranged from 83 to 100%.[9,17–18] Nevertheless, at present, follow-up review of glass slides remains an important aspect of quality assurance.

There are three types of telecytology systems: static, real-time (dynamic), and virtual (whole) slide imaging systems. Static image systems are cheaper, but they only allow the capture of a selected subset of microscopic fields. In static telecytology, preselected fields of view (FOV) (i.e. an image area produced by any camera and lens combination), captured as digital images, are forwarded to the consulting cytologist. Not surprisingly, several studies documented the time-consuming nature of acquiring digital images for static telecytology. Moreover, the digital image in static systems does not address adequacy of screening of the cytological material present on a slide. By comparison, the latter two telepathology systems permit evaluation of the entire slide, but they are costlier and may be hampered by high network traffic. With certain real-time telepathology systems, the consultant can actively operate a remote microscope with a robotic stage. Almost all of the telecytology studies have evaluated only static systems, to date. Further studies to evaluate the other telecytology systems are clearly needed.

Several limitations with regard to telecytology have been reported by investigators. In one study, for example, a higher cytotechnologist false negative rate for Pap tests was observed for telecytology, as compared to the light microscope.[11] Difficulties have included problems related to the slide (poor slide quality, low cellularity, poor staining), field selection (not representative, insufficient images), image quality (low resolution, out of focus, color quality), transmission, and interpretation. Even the most recent telecytology study from 2008 was limited by the use of black and white images with relatively poor resolution.[20] High resolution images and 24-bit true color images show better cellular detail, and can therefore be interpreted more accurately.[13] Image compression does not appear to hamper telepathology.[21] Some researchers found that their interpretation of telecytology images was hindered by their inability to examine cellular detail and to change the image focal plane, especially in thick and overlapping cell groups.[16,22] These factors may explain why some investigators had more difficulty with glandular abnormalities, as compared to squamous abnormalities.[19]

## VIRTUAL MICROSCOPY

Virtual microscopy (VM) or whole slide imaging (WSI) is the use of digital imaging to produce digital slides that simulate light microscopy. The entire slide is scanned and converted into a digital image. Virtual microscopy (VM) provides access to all areas of interest on a slide by using a computer or digital device, without the use of a microscope. In other words, the user can view a scanned image of the entire slide on a computer screen. Current systems are capable of complete, high speed digitization of slides, at multiple magnifications.[8] Selected scanning systems can even digitize multiple focal planes (x, y and z axes) to create a virtual slide with the ability to ‘focus’ at different magnifications. Current automated high-speed WSI systems are sufficient for diagnostic purposes and potentially represent a ‘disruptive technology’ in the traditional practice of pathology. A disruptive technology or disruptive innovation is a marketing term that refers to a technological innovation, product, or service that, when implemented, eliminates the existing dominant technologies (status quo) in a market, creates new markets, and/or drastically modifies markets.[23]

Whole slide imaging (WSI) is being used in surgical pathology for telepathology, consultation, archiving, clinical diagnosis, education, and examinations.[24] The application of VM in cytopathology has been limited to very few studies containing small numbers of cases.[25] This is surprising, as liquid-based specimens have smaller areas to scan, as compared to smears with the cellular material presented in a format typically more amenable to digital scanning, and are thus better suited for VM. Barriers to implementation of VM may include expensive initial setup, bandwidth restrictions, and the large file sizes of digitized slides. The one area of cytopathology where VM has made an appearance is proficiency testing.[26–30]

At present, manual screening and review of gynecologic cytology preparations is the current ‘gold standard’ for the assessment of proficiency. In certain countries such as the USA, the federal government has mandated a national proficiency testing program for gynecologic cytology. This demands that pathologists and cytotechnologists practicing in the USA undertake yearly testing using well-standardized glass slides. The use of digital images and computer-based methods has been proposed as an alternative to glass slides for proficiency testing, and has been shown to be a more cost-effective method.[29] Virtual microscopy (VM) allows participants to view digital images representing an entire cytologic glass slide at the same feature resolution currently available with light microscopy. The digital images are able to be stacked along a 3-dimensional z plane, allowing test participants to change focal planes (a key feature for the evaluation of cytologic material). However, technical and education/training advances such as improving the time required to examine digitized images are still required. For example, in one study, the individual performance based on glass slides was reported to have been better than the computer-based test.[28]

## COMPUTER SCREENING SYSTEMS

The Pap test has been remarkably successful as a cancer screening tool. However, as with every other medical technique or device, it is not perfect. The foundations of its design (a glass slide of smeared and stained cervical-vaginal cellular material to be reviewed by a trained human observer under light microscopy), with strengths in relative simplicity and low technical demands, have also proven to be at the root of its limitations. These limitations were recognized shortly after its widespread adoption in the 1950s, when initial efforts to automate Pap screening began. However, only as a result of significant technical and computer advances made in the past 10 to 15 years have computer screening systems been routinely utilized.[3] Apart from the USA, neural network-assisted primary screening has also been used on a large scale in Europe.[31]

In order to have accurate and efficient computer-assisted screening, the cellular material on the slide must be prepared in a standardized manner, conducive to rapid acquisition and computer processing. Problems related to thick conventional smears of exfoliated cells subject to variable air-drying artifact plagued the development of many early versions of automated screening devices during the 1950–1990 period. This was overcome with the fixation and processing of Pap tests via liquid-based cytology (LBC). Liquid-based cytology (LBC) for Pap tests came to fruition in the United States in 1996, when the Food and Drug Administration (FDA) approved the ThinPrep^®^ (Cytyc, a Hologic Company, Boxborough, MA, USA) as an alternative to the conventional Pap smear. This was followed by the approval of the AutoCyte Prep^®^, now BD SurePath^®^ (BD Diagnostics - TriPath, Burlington, NC, USA) in 1999, and, most recently, MonoPrep^®^ (MonoGen, Inc., Chicago, IL, USA) in 2006. Although each product is technically different in its approach, the final standardized result for each is a glass Pap slide, with its cellular component distributed in a relative monolayer, present over a reduced surface area, largely nonobscured by blood, inflammatory cells, and/or debris.

Automated laboratory instruments and screening systems have developed under two major system designs: (1) those that perform primary screening without cytotechnologist interaction, and (2) an interactive design that serves as the ‘cytotechnologist's cytotechnologist’, in which both the cytotechnologist and the computer depend upon each other for Pap test interpretation.

### 1. Primary screening systems

One of the major screening systems available today is an automated primary slide screener and archiver. The BD FocalPoint^®^ Slide Profiler, BD Diagnostics-TriPath (formerly AutoPap^®^ System, formerly AutoPap^®^ 300 QC System, NeoPath, Redmond, WA) is FDA (Food and Drug Administration-approved for both conventional smears and BD SurePath^®^ Pap tests. In the 1990s, the PAPNET^®^ System (Neuromedical Systems Inc., Suffern, NY) and the AutoPap 300 QC System were at the forefront of technological advances in the cervical cytology imaging race, each gaining FDA approval in the USA for the rescreening of previously manually screened conventional smears. These systems had the potential to greatly improve the yield of detecting false negative cases.[32–33] The AutoPap^®^ System subsequently altered its screening algorithm in the mid to late 1990s,[34–35] gaining FDA approval as a primary screening device in 1998 (with conventional smears) and in 2001 (with BD SurePath^®^ slides), and is currently known as the BD FocalPoint^®^ Slide Profiler. PAPNET, a QC system requiring screened glass slides to be sent to central review sites with scanning stations using adaptive computer processing (neural networks), produced images of pertinent cellular fields, which were then transferred to tape cassettes or CD-ROM to be returned to the cytology laboratory for review on high-resolution monitors.[36] It has since ceased to be marketed in the USA, largely due to the associated high costs per abnormal case detected and the overall logistical issues in its workflow design.[37–38]

The BD FocalPoint^®^ Slide Profiler is currently a self-contained unit, residing entirely on-site, within the cytopathology laboratory. Slides are scanned at varying objective levels, including high-resolution FOV scans of selected images, with computer processors assigning scores for each FOV based upon single cell, cell group, and thick cell group characteristics. These scores are then integrated into a final slide score, with each slide ranked (from 0 to 1.0) as to the likelihood that it may potentially contain a significant epithelial abnormality,[39] with the slides then being sorted into three major groups. Up to 25% of the successfully processed slides determined to have the lowest probability of containing abnormal cells (below the primary threshold) require no further review and can be directly reported as negative and archived (without human eyes examining the slide). The remaining 75% of the slides, designated as requiring human review, are further ranked in order of potential abnormality. The slide profiler also contains QC measures and checks, exceeding CLIA requirements of 10% random negative case rescreening by selecting 15% of the qualified negative cases for QC rescreen. It has the ability to automatically generate customizable work lists, slide sorting instructions, and result summaries, as well as the ability to identify inadequate Pap test samples.

### 2. Interactive screening systems

This screening design model relies on a close interaction between the computerized primary screener and the cytotechnologist (at a review microscope/station) in the screening interpretation of each Pap test. The major FDA-approved systems today are the ThinPrep Imaging System^®^ (Cytyc, a Hologic Company) and the BD FocalPoint^®^ GS (Guided Screening in concert with FocalPoint^®^ Slide Profiler) Imaging System (BD Diagnostics - TriPath). Each interactive screening system incorporates a system to scan slides, processes the cellular data using a host of predetermined (trained) cues in imaging algorithms, and drives cytotechnologist attention using automated X-Y axis relocation to cellular fields deemed significant, with the aid of an automated microscope, or review scope. The scanners and microscopes utilize slide barcodes, with engineered hardware devices incorporated into the microscopes, which include foot switch, mouse, automated stage, and key pad, enabling the cytotechnologist to readily maneuver through the initial review of the fields of interest, with electronic and physical marking capability, and if necessary, the full standard review of the slide. As a potential third entry into the interactive screening system market, MonoGen^®^ Inc. has recently announced that it has successfully completed proof-of-principle in its imaging effort and is now pursuing full product development.

With such interactive screening systems, the cytotechnologist benefits from improved overall job satisfaction,[40–41] decreased fatigue, and increased throughput (due to an approximately 70% reduction in the cellular surface area reviewed per negative slide).[42–43] This leads to an overall increased laboratory productivity, focused time spent on challenging cases, and attention directed to relevant fields of potential abnormality. The critical importance of human interpretation remains, as FOVs directed by the scanner rely on the diagnostic acumen of the reviewer.[40]

### 3. Screening system study findings

Table 2 includes a list of representative publications examining the efficacy of computer screening systems, performed over the last 13 years (1995-2008).[32,39–51] The performance of modern system designs has been largely positive, perpetuating continued system development and refinement, in addition to increasing clinical use in cytopathology laboratories worldwide. As was shown in most of these studies, as well as several abstracts that have been presented at cytology meetings in recent years (2004-2008),[52–63] the benefits derived from these screening systems include increased sensitivity of squamous intraepithelial lesion (SIL) detection, as a result of (a) accurate and sensitive quintile ranking of primary screening or interactive screening, (b) reduction in false negative cases, and/or (c) increased productivity. The increased throughput is of particular benefit in countries where cytotechnologists are not readily available. The limitations of computer-assisted screening has been largely ascribed to the ‘learning curve’ encountered when employing a new technology in clinical practice, and as this is, to a large degree, a human phenomenon, it has been primarily seen with interactive systems. Adverse findings in diagnostic performance, such as an initial increase in atypical squamous cells of undetermined significance (ASCUS) rates[40] or decreased human papilloma virus (HPV) positivity rates of ASCUS,[48] have largely been time-limited, with corrections noted in a relatively short time frame of months.

**Table 2:** Publications reporting the outcome with computer screening systems for Pap tests

*Reference*	*Date*	*Screening system primary/interactive*	*Major finding*
[32]	1995	Primary (AutoPap^®^)	Superior false negative detection to random rescreening
[39]	1999	Primary (AutoPap^®^)	HSIL placed in top three quintiles
[44]	2001	Primary (AutoPap^®^)	High sensitivity for review classification of LSIL+
[45]	2002	Primary (AutoPap^®^)	All cases of HSIL+ placed in top two ranking quintiles
[46]	2004	Primary (BD FocalPoint Slide Profiler^®^)	High % of HSIL placed in top two ranking quintiles
[47]	2005	Interactive (ThinPrep^®^ Imaging System)	Increased sensitivity for SIL detection
[48]	2006	Interactive (ThinPrep^®^ Imaging System)	Increased sensitivity for SIL detection, with biopsy confirmation of increased HSIL detection
[49]	2007	Interactive (BD FocalPoint GS^®^)	High sensitivity for SIL detection
[50]	2007	Interactive (ThinPrep^®^ Imaging System)	Increased sensitivity for SIL detection
[41]	2007	Interactive (ThinPrep^®^ Imaging System)	Increased sensitivity for SIL detection, with reduction of false negative rate; increase in cytotechnologist job satisfaction
[43]	2007	Interactive (ThinPrep^®^ Imaging System)	Increased productivity
[40]	2007	Interactive (ThinPrep^®^ Imaging System)	Increased sensitivity for SIL detection
[42]	2007	Interactive (ThinPrep^®^ Imaging System)	Increased productivity
[51]	2008	Interactive (ThinPrep^®^ Imaging System)	Unchanged ASCUS and HPV DNA positive rates

ASCUS = atypical squamous cells of undetermined significance, SIL = squamous intraepithelial lesion, LSIL = low grade SIL, HSIL = high grade SIL, + = higher diagnostic category

## ONLINE CYTOLOGY

The Internet is a global network of computer networks, which includes international telecommunications infrastructure. Internet functions include E-mail, the World Wide Web (web or www), and file transfer. HyperText Markup Language (HTML) used to create web pages facilitates the inclusion of digital images, in addition to text and links. Digital images on the Internet are typically available in Graphic Interchange Format (gif) image file format. GIF is a compressed bit-mapped format, designed to minimize file transfer time.

Information on the web has become increasingly useful to pathologists and has provided new opportunity for training, sharing research publications, and continuing medical education.[64–66] The Internet has also provided a mechanism for rural and underserved areas to gain access to healthcare, including cytology.[67]

Digital images have been used to develop several online cytology atlases, including the well known NCI Bethesda System Web Atlas.[68] This web-based atlas consists of 349 images representing a range of morphologic findings seen on both conventional smears and liquid-based preparations. For each image, the preparation type, morphologic criteria, and interpretation using the 2001 Bethesda System terminology are provided. A subset of these images was used for the web-based Bethesda Interobserver Reproducibility Study (BIRST), which involved over 500 participants providing independent interpretations online.[69] The resulting histograms showing the distribution of interpretations for these 77 images are presented on this website.

## IMAGE CYTOMETRY

Image cytometry is the image-based measurement of cells. Studies involving image cytometry allow very large populations of cells to be imaged and thereby analyzed. At present, the application of image cytometry to cytology is largely investigational. The most common current applications are for DNA analysis and the evaluation of immunohistochemical staining.[70] In digital image cytometry, once an image is acquired and objects of interest in the image have been selected (image segmentation), measurements can be made on them. This step, called feature extraction, leads to numerical data that can then be analyzed. Morphological assessment of cells by digital image analysis is believed to be objective and thus highly reproducible.[71] Static image analysis of the nuclear DNA content (as an indication of aneuploidy) in cytological smears of the uterine cervix has been shown to help distinguish low-grade from high-grade dysplastic lesions.[72–73] In an attempt to improve the positive predictive value for high-risk HPV in primary screening, DNA ploidy was measured, by some researchers, on the same liquid-based sample, by image cytometry in cases showing discrepancies between cytology and HPV testing.[74]

With the use of quantitative image analysis, some authors have proposed Sheffield quantitative criteria in cervical cytology, to assist in the diagnosis and grading of squamous intraepithelial lesions.[75–76] These authors found that using nuclear-to-cytoplasmic (N/C) ratios avoided problems associated with variable changes in nuclear and cytoplasmic areas, as may occur between conventional and different commercial LBC preparations.[75] Such quantitative image analysis applied to the British Society for Clinical Cytology (BSCC) quantitative criteria, to assist diagnosis in a three-tier grading system of squamous cell dyskaryosis, showed that the BSCC area N/C ratio criteria of grading squamous cell dyskaryosis required amendment. In addition, this study supported the new BSCC recommendation of low-and high-grade squamous cell categories, similar to the two-grade Bethesda System (TBS) for reporting squamous intraepithelial lesions.[76] Further studies in the field of image cytometry are anticipated.

## CONCLUSIONS

Digital images have ushered in the field of virtual pathology, that being the practice of diagnostic pathology in which an analytical technique is performed at one location and the necessary elements are transmitted in electronic form to another site for diagnostic interpretation. The advantages of digital images include potentially eliminating the need for glass slides (at least at the point of examination), allowing annotation to be added to images, and the ability to rapidly transmit and remotely share images electronically for several purposes (telecytology, conferences, education, quality assurance, peer review). In the field of cytology, digital images are currently utilized in telecytology, automated screening of Pap test slides, training and education (e.g. online digital atlases), and innovative techniques such as image cytometry. Additionally, significant potential exists for the use of digital images in gynecologic proficiency testing, irrespective of whether a test format persists or a continuing education set-up is adopted in the future. We predict that in the immediate future, digital images in cytopathology will likely be utilized for the rapid retrieval and review of previously imaged archived cases (e.g. digital libraries), telecytology of on-site rapid diagnostic interpretations for specimen triage (with regard to both the potential need for specialized processing/testing and the required level of morphology-based diagnostic expertise), screening of nongynecologic specimens processed by liquid-based techniques (e.g. urine and body cavity specimens), and for communication (e.g. teleconferences), and they will undoubtedly continue to play an ever-increasing role in training, education (particularly international), primary certification examinations, and maintenance of proficiency/certification. Ultimately, all screening and final interpretation/diagnosis of cytologic specimens (in addition to histologic/surgical material) may be performed at the computer screen rather than the light microscope.

Automation in the cytology laboratory is critical to meet current and future challenges. These include growing workloads, shortage of skilled cytotechnologists, and subspecialty centralization. However, while some of these concerns (growing workload and shortage of cytotechnologists) are currently applicable in some parts of the world, the opposite concerns have been most recently expressed.[77–78] With the prospect of primary HPV screening, HPV vaccine development, and extension of the recommended screening interval in select patient subsets, the future total volume of Pap tests may be significantly reduced. Remote interpretation of automated device-selected digitized images transmitted over the Internet has the potential to provide effective screening and clinical triage to individuals in underserved populations. Multispectral image analysis is an emerging tool that utilizes both spatial and spectral image information to classify images that can be used for the differentiation between benign and malignant cells. Such image analysis is currently being explored to detect malignancy in aspiration specimens.[79–80]

Compared to radiology, where the need for film was eliminated by digital imaging, in cytopathology the diagnostic preparation of material, at present, relies on glass slides. Cytologic material on glass slides needs to be converted into digital images by imaging or scanning the slide. Therefore, virtual slides currently increase the cost of rendering a diagnosis and these costs should be justified by an increase in convenience, speed, or accuracy of diagnosis. Moreover, virtual slides at present have not been uniformly accepted by cytotechnologists and cytopathologists.[81] With current available technology, if imaged in multiple planes slides would take a relatively long time to image and result in large file sizes that may preclude speedy transmission and increase data storage costs. It is also of note that occasional published papers on imaging systems suggest that they may, in fact, have limitations in detecting koilocytes.[82] Furthermore, limitations and diagnostic errors related to telecytology have been linked by several investigators to the misinterpretation of digital images, leading some authors to actually recommend refinement of diagnostic cytologic criteria for digital images.[14–17] On the other hand, additional cytology studies are required to evaluate more advanced telepathology systems currently available, including several systems that now have the ability to change focal planes. Cytologists in Europe are at a particular advantage to test these technologies, as regulatory agency oversight appears to be less stringent than in the USA and many European countries have a national patient identification number and/or pathology data archive. The rapidity of scanning entire slides with current whole slide imagers makes digital imaging more amenable to rapid immediate interpretations of aspirated material. However, validation of digital imaging systems and their uses, reimbursement, and medicolegal issues surrounding telepathology still need to be refined.[83–84] Moreover, standardization of the entire imaging process is necessary, especially given that alteration of digital images may result in significant diagnostic misinterpretation.[85]

Finally, it should be pointed out that while LBC was designed with a major (if not primary) intent of enabling computer screening devices, this technology has generally been shown to improve and standardize the overall specimen quality, reduce unsatisfactory Pap test rates, and improve the rates of detection of significant and potentially significant cervical-vaginal lesions. In addition, the residual liquid vial sample has seemingly endless potential for use in ancillary molecular studies, above and beyond the morphologic Pap test, such as HPV DNA testing. As automated interactive computer screening continues to improve Pap test sensitivity, the evolving role of the ‘digital’ Pap test warrants re-examination.

## COMPETING INTEREST STATEMENT BY ALL AUTHORS

No competing interest to declare by any of the authors.

## AUTHORSHIP STATEMENT BY ALL AUTHORS

Each author acknowledges that this final version was read and approved.

According to International Committee of Medical Journal Editors (ICMJE http://www.icmje.org) “author” is generally considered to be someone who has made substantive intellectual contributions to a published study.

Authorship credit should be based on 1) substantial contributions to conception and design, acquisition of data, or analysis and interpretation of data; 2) drafting the article or revising it critically for important intellectual content; and 3) final approval of the version to be published. Authors should meet conditions 1, 2, and 3. Other contributors, who do not meet these criteria for authorship, are listed in an acknowledgments section.

All authors of this article declare that we qualify for authorship as defined by ICMJE http://www.icmje.org/#author.

Each author has participated sufficiently in the work and take public responsibility for appropriate portions of the content of this article.
